# Effectiveness of a programable body-worn digital hearing aid for older adults in a developing country: a randomized controlled trial with a cross-over design

**DOI:** 10.1186/s12877-021-02325-4

**Published:** 2021-07-23

**Authors:** Pornthep Kasemsiri, Kwanchanok Yimtae, Panida Thanawirattananit, Pasin Israsena, Anukool Noymai, Supawan Laohasiriwong, Patravoot Vatanasapt, Pipop Siripaopradith, Pritaporn Kingkaew

**Affiliations:** 1 Department of Otorhinolaryngology, Faculty of Medicine, Khon Kaen University, Khon Kaen, 40002 Thailand; 2 Khon Kaen Ear Hearing and Balance Research Group, Khon Kaen University, Khon Kaen, Thailand; 3 National Electronics and Computer Technology Center (NECTEC), National Science and Technology Development Agency (NSTDA), Prathumthani, Thailand; 4 Phuwieng Hospital, Ministry of Public Health, Khon Kaen, Thailand; 5 Health Intervention and Technology Assessment Program (HITAP), Ministry of Public Health, Nonthaburi, Thailand

**Keywords:** Hearing aid, Hearing loss, Older adults, Rural community

## Abstract

**Background:**

Hearing aids are important assistive devices for hearing rehabilitation. However, the cost of commonly available commercial hearing aids is often higher than the average monthly income of individuals in some developing countries. Therefore, there is a great need to locally produce cheaper, but still effective, hearing aids. The Thai-produced P02 hearing aid was designed to meet this requirement.

**Objective:**

To compare the effectiveness of the P02 hearing aid with two common commercially available digital hearing aids (Clip-II™ and Concerto Basic®).

**Methods:**

A prospective, randomized controlled trial with a cross-over design was conducted from October 2012 to September 2014 in a rural Thai community. There were 73 participants (mean age of 73.7 ± 7.3 years) included in this study with moderate to severe hearing loss who were assessed for hearing aid performance, including probe microphone real-ear measurement, functional gain, speech discrimination, and participant satisfaction with the overall quality of perceived sound and the design of the device.

**Results:**

There were no statistically significant differences in functional gain or speech discrimination among the three hearing aids evaluated (*p*-value > 0.05). Real-ear measurements of the three hearing aids met the target curve in 93% of the participants. The best real-ear measurement of the hearing aid following the target curve was significantly lower than that of Clip-II™ and Concerto Basic® (*p*-value < 0.05) at high frequency. However, participants rated the overall quality of sound higher for the P02 hearing aid than that of Clip-II™ but lower than that of Concerto Basic**®** (*p*-value > 0.05). Participants revealed that the P02 hearing aid provided the highest satisfaction ratings for design and user-friendliness with statistical significance (*p*-value < 0.05).

**Conclusion:**

The P02 hearing aid was an effective device for older Thai adults with hearing disabilities. Additionally, its modern design, simplicity of use, and ease of maintenance were attractive to this group of individuals. These benefits support the rehabilitation potential of this hearing aid model and its positive impact on the quality of life of older adults in developing countries.

**Trial registration:**

This study was registered under Clinicaltrial.govNCT01902914. Date of registration: July 18, 2013.

## Background

Hearing impairment is a global problem that affects communication and individuals’ quality of life. In 2019, the World Health Organization (WHO) estimated that 1.57 billion people globally presented with hearing loss. Of all people with hearing impairment, 62.1% were older than 50 years. With an ageing society, the number of people with hearing loss will increase to an estimate of 2.45 billion people by 2050 [1]. Furthermore, the prevalence of hearing impairment is higher in low- and middle-income countries than in high-income countries [2]. Presbycusis is the common cause of hearing loss worldwide [3]. The exact prevalence of presbycusis is difficult to determine due to the different criteria used to define hearing loss; however, Wattamwar et al. [4] estimated that presbycusis affects more than half of older adults by age 75 years and nearly all adults over age 90 years. Hearing impairment negatively impacts personal health [5] and aspects of living, including communication, socialization, and safety; therefore, hearing-impaired patients, especially older adults, may have increased social isolation and decreased autonomy [6]. Furthermore, hearing loss may influence aspects of mental well-being, such as anxiety, depression, and lethargy [7, 8]. Uhlmann et al. also reported that hearing impairment is related to dementia and cognitive dysfunction in older adults [9]. Several studies have shown that age-related hearing loss is associated with an increased risk of developing dementia [10, 11]. Therefore, aural rehabilitation is essential in the management of age-related hearing loss to prevent and relieve the consequences that have negative effects on a person’s quality of life.

Hearing aids play an important role in aural rehabilitation; however, the cost of hearing aids is higher than the average monthly income of some individuals in Thailand [12]. The WHO has estimated that the number of hearing aids produced is less than one-tenth of that needed, and three-quarters of these devices are distributed in North America and European countries. One-quarter of these devices are distributed throughout the rest of the world, with half of these being distributed in high-income countries. Therefore, the WHO has also urged developing countries to produce their own hearing aids or to import a large volume of low-cost hearing aids to increase the accessibility of these devices to persons with hearing disabilities [13].

**Fig. 1:**
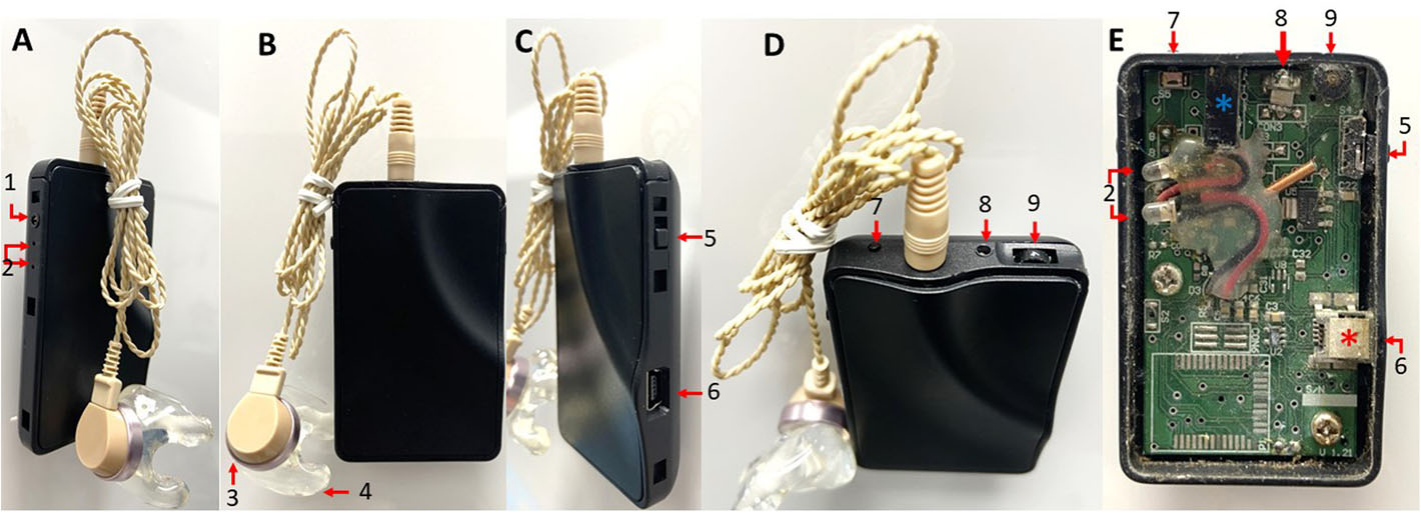
**A**–**E** The P02 is a digital programmable body-worn hearing aid. Its size dimensions are 65 mm × 45 mm × 15 mm, it has a built-in rechargeable battery, and its total weight is 25.7 g. The P02 device consists of a channel for programming the adjustment system (1), a battery capacity indicator (2), an ear receiver (3), an ear mould (4), an on/off switch (5), a channel charger (6), a battery compartment (red asterisk), a programme button (7), an amplifier speaker (blue asterisk), a microphone (8), and a volume control (9). The P02 has a 2-channel wide dynamic range compression with 5-band equalizer hearing aids. It has 4 memory slots with multi-memory tone indicators. Digital signal processing in the P02 provided sound with a maximum amplification output of 123 dB and an average peak gain of 66 dB. Regarding the occlusion effect, we adjusted the low-frequency gain and modified the ear mould with venting following an adjustment to the individual hearing threshold level

The National Electronics and Computer Technology Center (NECTEC), a Thai governmental organization, developed body-worn aid, digital, programmable hearing aids and has been producing them since 2006. The first model, PDN-01B, also called P01, met the electro-acoustical test standards set by the International Electrotechnical Commission (IEC 60118–7) [14]. Clinical testing revealed that users were very satisfied at both 3, and 6 months regarding ease of communication and speech understanding in a moderately reverberant room and other environments with competing noise. Hence, the P01 model indicated suitable for users with moderate to severe hearing impairment [14]. P01 was later modernized, giving it a similar look to a media player (model P02) (Fig. 1A–E). This design with modified and more prominent buttons and wheel volume control aimed to facilitate use by older adults, and its modern design encouraged older adults to wear the hearing aid. The P02 battery was changed to a rechargeable lithium ion battery from the zinc-air batteries in the P01 model.

This study was designed to compare the effectiveness of a locally produced Thai body-worn hearing aid (P02) versus two common commercially available digital trimmer hearing aids, Concerto Basic**®** (Beltone Electronics Corp., Denmark) and Clip-II**™** (GN ReSound A/S., Denmark). These three hearing aids have a similar level of amplification. Their specifications are provided in Table 1.

**Table 1:** Comparison characteristics of the hearing aids

Characteristics	P02	Concerto basic	Clip-II™
Gain adjustable by pre-set options or user controls	Programmable	Screw trimmer	Screw trimmer
Electroacoustic specification (IEC118–7 2 cc. Coupler)	Digital	Digital	Digital
Maximum output (OSPL90) 118 ± 4 dBSPL	123 dB SPL	129 dB SPL	129 dB SPL
Maximum output at 1 KHz (OSPL90) 114 ± 4 dB	117 dB SPL	121 dB SPL	121 dB SPL
Maximum FOG (45–55 dB + 5 dB)	66 dB SPL	67 dB SPL	67 dB SPL
Maximum FOG at 1 KHz (42 + 5 dB)	63 dB SPL	64 dB SPL	64 dB SPL
Basic frequency range 200–4500 Hz	573–4400 Hz	100–3990 Hz	130–3690 Hz
Total dynamic distortion 500 Hz < 5%	0.6%	2.6%	NA
Total dynamic distortion 800 Hz < 5%	3.8%	2.1%	2.1%
Total dynamic distortion 1600 Hz < 2%	0.4%	0.1%	0.1%
Equivalent input noise level < 25 dB SPL	31.4 dB SPL	24 dB SPL	24 dB SPL
Powered by zinc-air or rechargeable	Rechargeable Lithium-ion	Zinc-air models	Zinc-air models
Battery current	11.8 mA	0.65 mA	0.65 mA
Maintenance	Charged by electricity for 3 h every 3–4 days	Changing the battery every month	Changing the battery every month
Price	Estimated 100 USD	300 USD	300 USD

## Methods

A prospective, randomized controlled trial with a cross-over design was conducted from October 2012 to September 2014 in rural Thai districts, including Phuwieng; Wiengkao; and Nongnakum, Khon Kaen Province. Inclusion criteria were participants who were ≥ 60 years old, were new hearing aid candidates, had bilateral sensorineural hearing loss with an average pure-tone air-conduction threshold between 500 and 2000 Hz in their better-hearing ear within a range of 41–75 dB, had no otorrhea for at least 3 months, had no pathology of the external ear canal by otoscopy examination, and had no suggestive middle ear effusion or mass by tympanometry. Participants were excluded if they had speech discrimination with a Thai monosyllable word list of less than 40% in both ears or suggestive retro-cochlear pathology. Written informed consent was obtained from all participants.

Participants tested all three hearing aids with the P02 device considered the intervention and Concerto Basic**®** and Clip-II**™** devices considered as the controls. The testing order of the three hearing aids was produced by computer-generated randomization for each participant. The audiologists were blinded to the generated order sequence since each participant’s order was placed in an envelope before the hearing aid testing began. All participants’ hearing levels were tested with a standard audiogram before the hearing test day and on the same day as the experiment; hearing aid testing was performed on the better hearing ear. Hearing aid performance was evaluated by probe microphone real-ear measurement and was adjusted for each participant, and each hearing aid met the best target curve by an audiologist in a quiet room. The hearing aid was then placed in a bag for masking from the second audiologist and participants. In a standard soundproof room, participants’ functional gain and speech discrimination before and after using each hearing aid were evaluated by a second audiologist to avoid bias. Participants and the second audiologist were blinded to the brand of hearing aid and the hearing aid performance results.

After each participant used the three hearing aids, the participant was asked to choose the top two ranks of hearing aids in order of preference according to their overall satisfaction with the quality of perceived sound. However, the participant was able to choose more than one brand of hearing aid in the same ranking.

The participant was still blinded to the hearing aid identity during this choice task. In addition, participants were asked if they were satisfied with the hearing aid design by another researcher who was blinded to the hearing aid data. The ranking of satisfaction in the hearing aid design was evaluated in the same fashion as the satisfaction of quality sound perception.

The primary hearing aid performance outcome was the functional gain in speech frequency, analysed by subtracting the unaided and aided air-conduction threshold and pure-tone average threshold at 500–2000 Hz in the free field. Real-ear measurement, speech discrimination score, quality of sound, and design satisfaction were considered secondary outcomes. Paired *t*-tests using 95% confidential intervals were used to compare the mean differences. The chi-square test was used to test ordinal variables. A value of *p* < 0.05 was considered statistically significant. This study was registered under Clinicaltrial.gov (NCT01902914), and the protocol was reviewed and approved by the Human Ethics Committee of Khon Kaen University (HE551268).

## Results

**Fig. 2:**
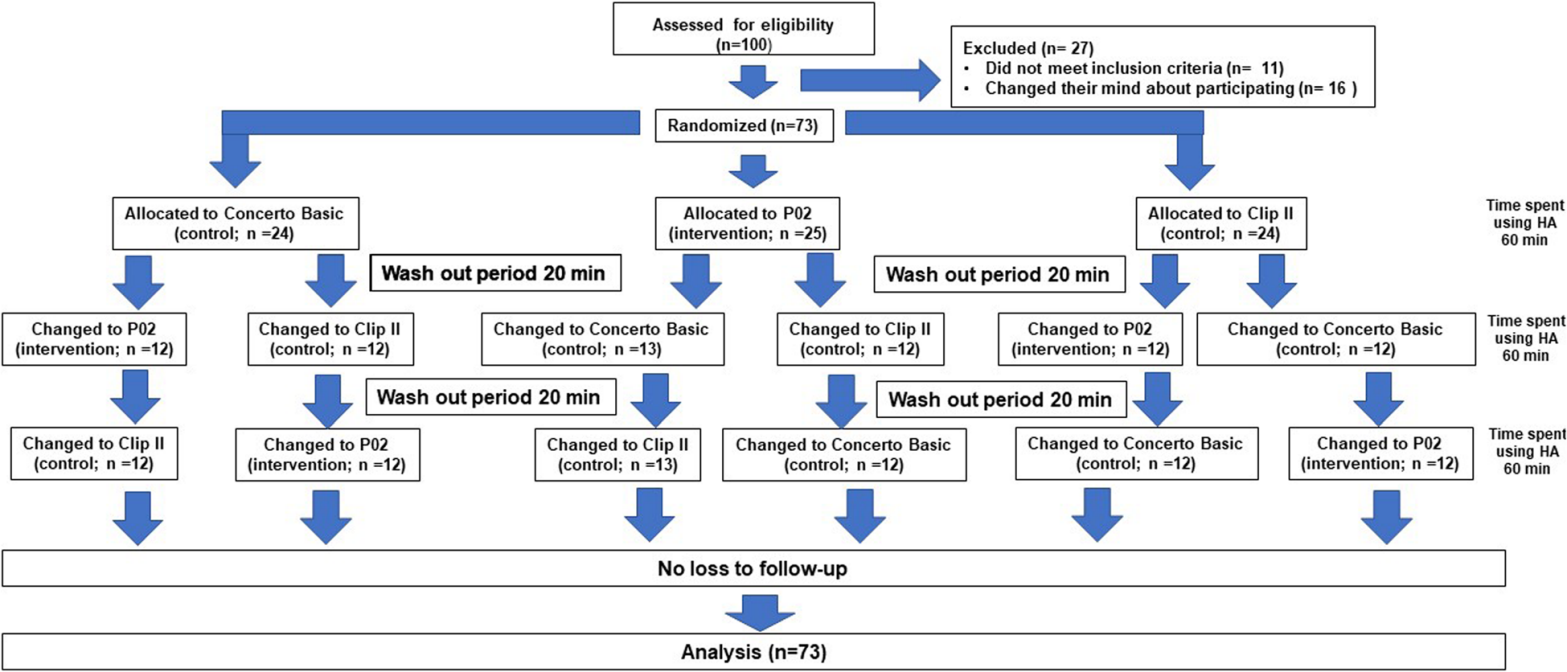
The research study protocol

One hundred eligible people initially consented to participate in the study. However, 16 withdrew from the study before visiting the audiology clinic on the day of the proposed hearing aid evaluation. Thus, 84 participants underwent a repeat examination by otologists and audiology testing to confirm that they met the eligibility criteria; 11 participants (6 that were suggestive of middle ear pathology, 4 with hearing levels that did not reach study criteria, and 1 with a discrimination score worse than 40%) were then excluded. Thus, 73 participants eventually completed the full study protocol presented in the CONSORT diagram (Fig. 2).

**Fig. 3:**
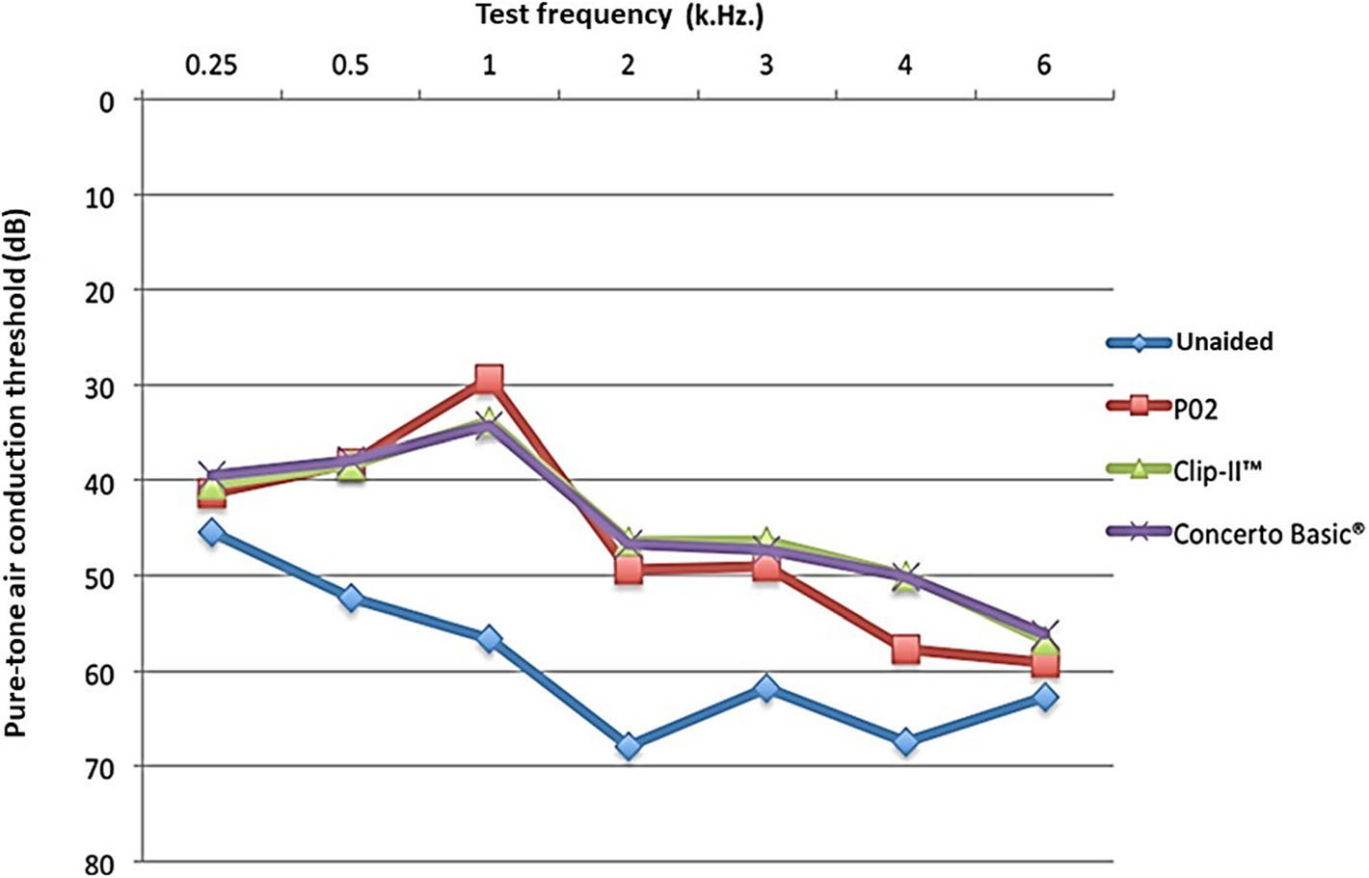
The mean air-conduction pure-tone aided and unaided thresholds for each frequency across the Clip-II™**,** the Concerto Basic**®**, and P02 hearing aids

All participants self-reported hearing loss, with 63% reporting tinnitus and aural pressure and 34% reporting vertiginous symptoms. The demographic data is shown in Table 2. The mean unaided pure-tone average threshold of the fitting ear was similar, although it was analysed according to the different criterion guidelines, including the American Speech-Language-Hearing Association (ASHA), American Academy of Otolaryngology-Head and Neck Surgery (AAO-HNS), and World Health Organization (WHO) (Table 2). Functional gain and speech discrimination were not significantly different across the three hearing aids (Table 3). The mean air-conduction pure-tone aided and unaided thresholds of each frequency are displayed in Fig. 3.

**Table 2:** Demographic data

Characteristics	Values	95% CI
Sex (n)		
Male	46 (63.01%)	51.55–73.18
Female	27 (36.99%)	26.82–48.45
Mean age ± SD	73.67 ± 7.23 years	71.98–75.36
Range of age	60–92 years	
Air-conduction PTA (500–2000 Hz)		
Rt ear	58.84 ± 14.84 dB	55.38–62.30
Lt ear	57.64 ± 10.76 dB	55.13–60.15
Mean unaided PTA of fitting ear ± SD		
500–2000 Hz (ASHA)	58.92 ± 7.42 dB	57.19–60.65
500–3000 Hz (AAO-HNS)	59.63 ± 7.57 dB	57.86–61.39
500–4000 Hz (WHO)	61.05 ± 7.67 dB	59.26–62.84

**Table 3:** Comparison of functional gain and speech discrimination among the three hearing aids

**Hearing aids**	**P02**	**Clip-II™**	**Concerto basic®**
Average functional gain (dB)	20.14 ± 6.23 (95% CI: 18.66–21.54)	19.41 ± 5.40 (95% CI: 18.15–20.65)	19.44 ± 5.43 (95% CI: 18.15–20.65)
Average speech discrimination (%)	67.8 ± 17.13 (95% CI: 63.80–71.79)	67.6 ± 18.13 (95% CI: 63.37–71.83)	68.8 ± 17.91 (95% CI: 64.62–72.98)
**Comparison of hearing aids**	**P02 VS Clip-II™**	**P02 VS Concerto Basic®**	**Clip-II™ VS Concerto Basic®**
Mean difference of functional gain	0.73 ± 4.08 (95% CI: − 0.22 - 1.68)	0.70 ± 4.20 (95% CI: − 0.28 - 1.68)	0.03 ± 2.84 (95% CI: − 0.63 - 0.69)
*P*-value	0.13	0.16	0.93
Mean difference of speech discrimination	0.22 ± 6.7 (95% CI: − 1.55 - 1.99)	1.00 ± 6.45 (95% CI: − 0.51 - 2.50)	1.22 ± 6.43 (95% CI: − 0.28 - 2.72)
*P*-value	0.78	0.19	0.11

We found that the P02 mean air-conduction pure-tone aided and unaided thresholds were significantly better than those of the Clip-II™ and the Concerto Basic**®** at 1000 Hz (*p*-value < 0.05); conversely, the Clip-II™ and the Concerto Basic**®** performance at 4000 Hz was better than that of the P02 (*p*-value < 0.05).

**Fig. 4:**
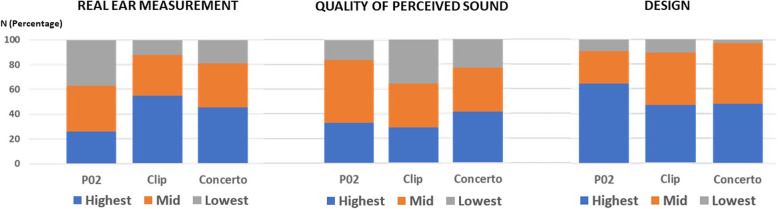
Comparison of hearing aid performance

In objective real-ear measurement testing, the three hearing aids met the target curve in 93% of the participants for each hearing aid. Subgroup analysis showed that the P02 real ear measurement was farther from the target curve than that of the Clip-II™ and that of the Concerto Basic**®** at a frequency of more than 2000 Hz; the best closest objective real-ear measurement to the target curve of P02 was lower than those of the Clip-II™ and the Concerto Basic® (*p*-value < 0.05). However, participants’ subjective assessment of overall sound quality showed a preference for the P02 device over the Clip-II™ but was lower than that of the Concerto Basic**®** (*p*-value > 0.05). Participants’ satisfaction with the hearing aid design was the highest for the P02 (*p*-value < 0.05) (Fig. 4**)**.

## Discussion

Many factors affect hearing aid selection, including the degree of hearing loss, problems experienced by the person, patient motives and expectations, personality traits, auditory counselling, and economic issues [15]. Furthermore, the period of hearing aid acclimatization is the other factor that may affect one’s choice in hearing aid. Unfortunately, in Thailand, the current practice is that hearing aid trials are done on the same day with hearing aid fitting. This is not ideal but is conducted according to government policies of hearing aid testing, limiting the number of tested hearing aids and travel expenses that burden the patient if they were to return for each test. Therefore, our study was designed according to current practice, and the period of hearing aid acclimatization became short. However, the follow-up to adjust the hearing aid was continued after fitting until the rehabilitation goal was reached.

The price of a hearing aid is one of the barriers against patient use; therefore, locally produced hearing aids with low cost would minimize this obstacle while maximizing its coverage in low- and middle-income countries. Recently, several designs of hearing aids suitable for this purpose have been made available. The body-worn hearing aid is the largest one that may be more convenient (for elderly individuals) as they are easier to see and manipulate [16]. Many older adults with hearing impairment may have comorbidities, including impaired vision, limited touch sensation and range of movement, and dementia [17–22]; therefore, a small area behind the ear or on the ear hearing aid may lead to increased management issues for older adults [17]. Several studies show that older adult hearing aid users have difficulties in basic hearing aid management, including correctly inserting the aid or adjusting volume controls [23–25]. A body-worn design is less commonly used in developed countries. Taylor et al. reported that body-worn hearing aids comprised less than 1% of the hearing aid market [26]. This small market share limits choices for selection of the body-worn hearing aids with proper cost and suitability for older users’ lifestyles in our country; therefore, the P02 model, a Thai manufactured digitally programmable body-worn hearing aid, was designed to suit older adult users’ lifestyles more appropriately. This hearing aid provides older users with several benefits, such as greater electroacoustic flexibility, easier volume control management, multiple programmes, and faster fitting.

In our study, functional gain and speech discrimination with the P02 device were found to be similar to those obtained with Clip-II**™** and Concerto Basic**®**. However, Concerto Basic® and Clip-II™ provided a significantly better functional gain than the P02 device at 4000 Hz, whereas the functional gain of the P02 device was significantly better than that of Concerto Basic® and Clip-II™ at 1000 Hz. Although these differences reached statistical significance, differences less than 10 dB may have a minor impact on hearing in clinical practice. Regarding objective real-ear test performance, Concerto Basic® and Clip-II™ were better than the P02 model at high frequencies. Notably, participants’ subjective satisfaction ratings of overall sound quality were higher for the P02 device than for both alternatives. These results may be due to the different techniques utilized to limit excessive amplifier sound across hearing aids. Clip-II™ and Concerto Basic**®** use linear peak clipping, whereas the P02 model uses a wide dynamic range compressor. Both limiters produce some sound distortion and loss of sound detail; thus, the result of the real-ear test showed that the curves of the three hearing aids were different from the target curve, with the P02 curve being furthest apart at the highest frequency. Noffsinger et al. [27] have previously shown that the wide dynamic range compressor produces a clearer and more comfortable sound, likely reflected in the participants’ higher satisfaction with the P02 device regarding overall sound quality over either the Clip-II™ or Concerto Basic**®**.

The P02 design, similar to a modern music media player rather than appearing as a disability aid, reduces stigma for the wearer. Undoubtedly, this positive attitudinal feature contributed to its higher satisfaction rating compared with the other two aids. The P02 is a lighter-weight hearing aid with a built-in rechargeable battery, holds a 3-day charge, and is easily charged by a main electricity supply. Using rechargeable batteries is more convenient than having to regularly replace disposable batteries and reduces electronic waste. The P02 hearing aid is still in the prototype phase, but the cost is estimated to be 100 USD, which is cheaper than currently available commercial aids; thus, it is more affordable for older adults in a rural community in developing countries.

In summary, the P02 device seemed as effective as Clip-II™ and Concerto Basic**®**, the commonly available commercial hearing aids, although participants gave the P02 model higher subjective ratings for quality of sound. A P02 limitation is the detail distortion that individuals experienced in high frequencies, as shown in the real-ear result. Any adjustments to obtain more gain in the high-frequency range should maintain the same comfortable sound. A limitation of this study is the short time that individuals spent using hearing aids to acclimatize to them; therefore, a longer duration of use of the hearing aids would be useful to assess satisfaction with the device.

## Conclusion

The P02 model, a Thai-produced digital programmable body-worn hearing aid, seemed as effective as two other comparable common commercial hearing aids for use with older Thai adults with hearing disabilities. Furthermore, the P02 device has the benefits of a modern design, simplicity of use, potential cost savings, and maintenance convenience via the use of a built-in rechargeable battery.

## Data Availability

The protocol, datasets used and/or analysed during the current study are available from the corresponding author on reasonable request.

## Abbreviations

WHO: World Health Organization
NECTEC: The National Electronics and Computer Technology Center
IEC: International Electrotechnical Commission
ASHA: American Speech-Language-Hearing Association
AAO-HNS: American Academy of Otolaryngology-Head and Neck Surgery
